# How to apply an eye pad, shield, and bandage

**Published:** 2010-12

**Authors:** Sue Stevens

**Affiliations:** Former Nurse Advisor, Community Eye Health Journal, International Centre for Eye Health, London School of Hygiene and Tropical Medicine, Keppel Street, London WC1E 7HT, UK.

**Figure:**
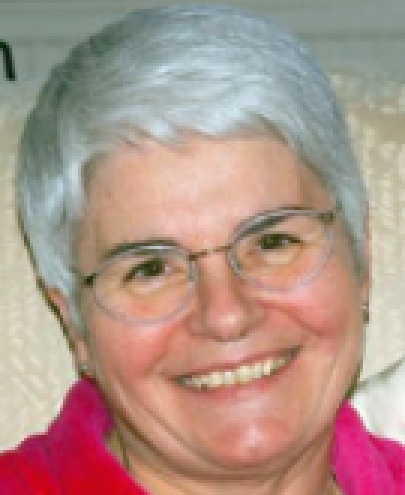


Before performing any eye procedure:

- **Wash your hands** (and afterwards too).
- **Position the patient comfortably** with head supported.
- **Avoid distraction** for yourself and the patient.
- Ensure **good lighting.**
- Always **explain to the patient** what you are going to do.

## Eye pad

**Reasons for applying an eye pad**

- to **‘rest’ the eye**
  e.g., hyphaema, vitreous haemorrhage.
- to **aid healing following trauma**
  e.g., corneal abrasion.
- to **protect the eye**
  e.g., following surgery and procedures requiring corneal anaesthesia.

**You will need**

- eye pad
- eye shield
- scissors
- adhesive tape

**Preparation**

It is important to remind the patient not to open the affected eye under the pad. If the eyelids do not close naturally over the cornea it will be necessary, before padding, to tape the eyelids closed.

**Figure 1:**
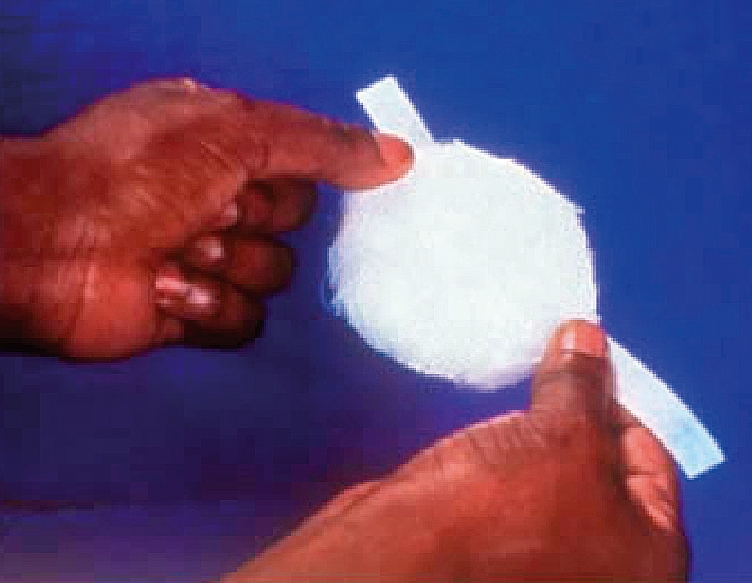


**Method**

**Figure 2:**
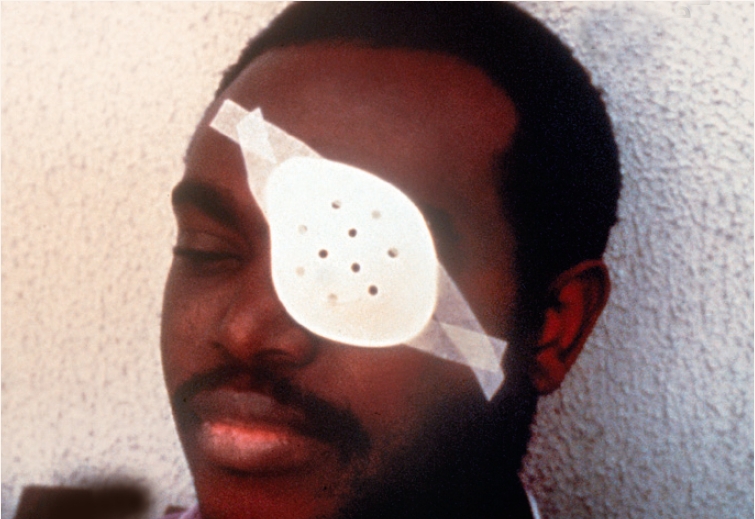


1. Apply a piece of adhesive tape, about 15 centimetres long, to the eye pad (Figure 1).
2. Ask the patient to close both eyes.
3. Position the eye pad diagonally over the closed lids of the affected eye and tape firmly, but gently, to the forehead and cheek.
4. Apply a second and third piece of tape to ensure the pad lies flat.
5. Extra protection can be given by taping a shield over the pad in the same way. The shield shown (Figure 2) is produced commercially and is called a Cartella shield. You can also make your own (see box).

## Eye bandage

**Reasons for applying an eye bandage**

To maintain gentle pressure over an eye pad:

- to arrest haemorrhage
- to reduce swelling after eyelid surgery
- following eye surgery, e.g., enucleation
- for a child, to ensure the pad is not disturbed.

**You will need**

- bandage – 5 centimetres wide
- eye pad
- adhesive tape
- small safety pin

**Method**

**Figure 3:**
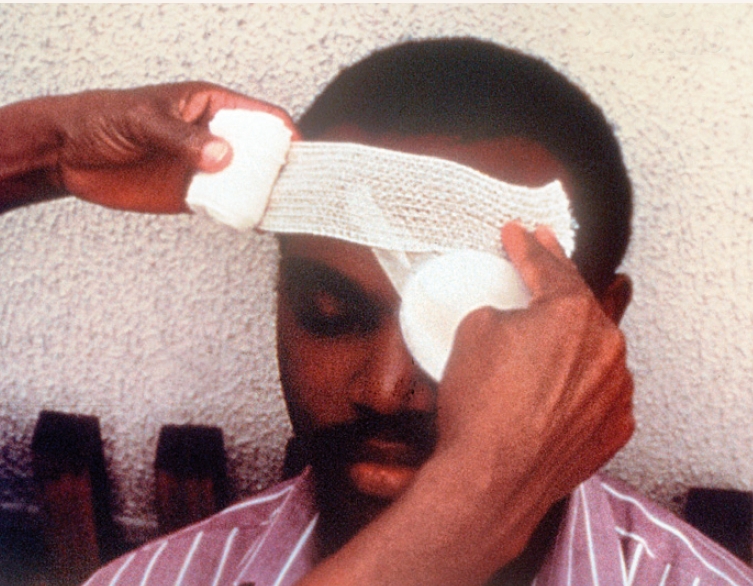


**Figure 4:**
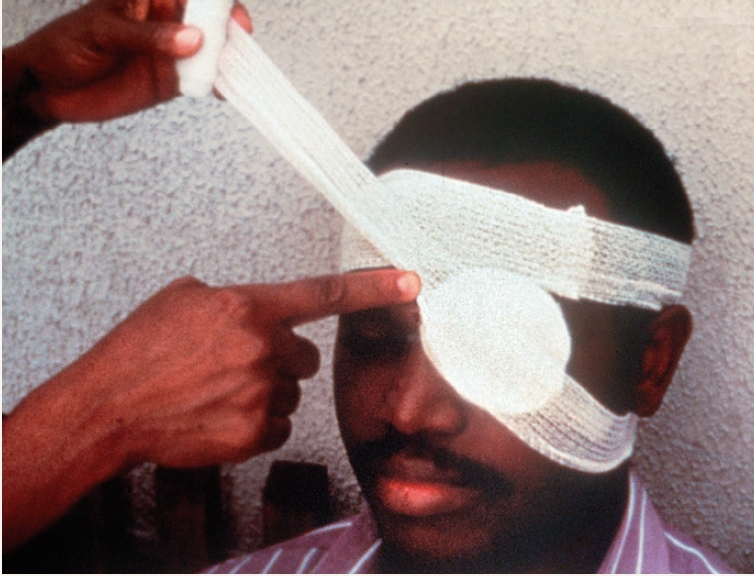

1. Apply an eye pad as described above.
2. Hold the rolled bandage in one hand with the opened end, held by the other hand, on the forehead above the affected eye (Figure 3).
3. Take the bandage, directed away from the affected eye, twice around the head firmly, but not tightly.
4. On the second circuit, bring the bandage below the ear and up over the eye and around the head again.
5. The bandage can partially obscure the other eye. To avoid this happening, place the index finger above the eyebrow and hold up the edge of the bandage (Figure 4).
6. Continue the two circuits described above until the bandaging is complete.
7. Secure with adhesive tape and/or small safety pin (but do not use a pin in the case of a child).

**Making an eye pad and eye shield**

**You will need**

- cotton wool
- two pieces of gauze
- scissors
- adhesive tape
- thin cardboard or old X-ray film
- circular object - about 8 centimetres in diameter
- pencil

**Figure 5:**
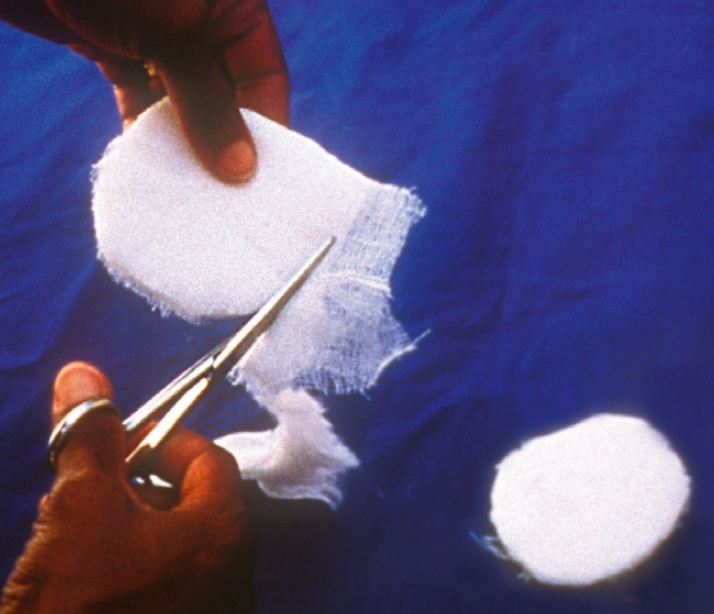

**How to make an eye pad**

1. Place cotton wool between the two pieces of gauze.
2. Cut into an oval shape approximately 5 centimetres wide and 6 centimetres long (Figure 5).

**Figure 6:**
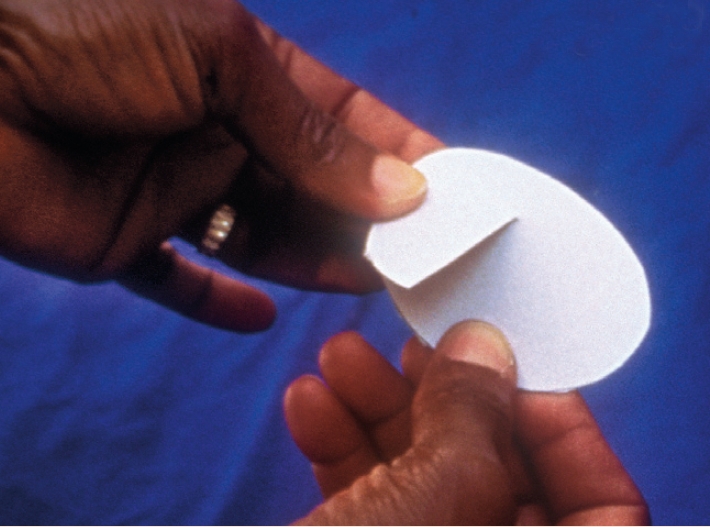

**How to make an eye shield**

1. Draw a circle on the cardboard or film and cut around it.
2. Make a single cut into the centre (just half the diameter).
3. Turn into a cone (Figure 6) and secure the shape with adhesive tape.

